# Functional Characterization of Stromal Osteopontin in Melanoma Progression and Metastasis

**DOI:** 10.1371/journal.pone.0069116

**Published:** 2013-07-23

**Authors:** Santosh Kumar, Priyanka Sharma, Dhiraj Kumar, Goutam Chakraborty, Mahadeo Gorain, Gopal C. Kundu

**Affiliations:** National Center for Cell Science (NCCS), NCCS Complex, Pune, Maharashtra, India; University of Colorado, United States of America

## Abstract

**Background:**

Recent studies demonstrated that not only tumor derived- but stroma derived factors play crucial role in cancer development. Osteopontin (OPN) is a secreted non-collagenous, sialic acid rich, chemokine-like phosphoglycoprotein that facilitates cell-matrix interactions and promotes tumor progression. Elevated level of OPN has been shown in melanoma patient and predicted as a prognostic marker. Recent reports have indicated that stroma-derived OPN are involved in regulating stem cell microenvironment and pre-neoplastic cell growth. However, the function of stroma derived OPN in regulation of side population (SP) enrichment leading to melanoma growth, angiogenesis and metastasis is not well studied and yet to be the focus of intense investigation.

**Methodology/Principal Findings:**

In this study, using melanoma model, in wild type and OPN knockout mice, we have demonstrated that absence of host OPN effectively curbs melanoma growth, angiogenesis and metastasis. Melanoma cells isolated from tumor of OPN wild type (OPN^+/+^) mice exhibited more tumorigenic feature as compared to the parental cell line or cells isolated from the tumors of OPN KO (OPN^−/−^) mice. Furthermore, host OPN induces VEGF, ABCG2 and ERK1/2 expression and activation in B16-WT cells. We report for the first time that stroma derived OPN regulates SP phenotype in murine melanoma cells. Moreover, loss in and gain of function studies demonstrated that stroma-derived OPN regulates SP phenotype specifically through ERK2 activation.

**Conclusions:**

This study establish at least in part, the molecular mechanism underlying the role of host OPN in melanoma growth and angiogenesis, and better understanding of host OPN-tumor interaction may assist the advancement of novel therapeutic strategy for the management of malignant melanoma.

## Introduction

Recent trends in cancer research are focused on understanding the complex crosstalk between tumor and stromal microenvironment. The progression and spread of the cancer cells from the site of origin to distant organ not only depends on the intrinsic factors produced by cancer cells but the stromal factors derived from host environment [1]–[5]. It has been hypothesized that tumor development depends upon the mutual interaction between the genetically altered malignant cells and the dynamic microenvironment in which they grow [1], [4]. Although the “seed and soil” hypothesis of cancer progression had been proposed by Paget more than hundred years ago, but till today the role of soil (stromal microenvironment) in cancer progression is not understood clearly as compared to the function of seed (tumor cell) in this process [6]. Therefore, to determine the role of host/stromal environment as well as stromal factors in the development of tumor malignancies not only helps in understanding the molecular mechanism of cancer progression but may also spawn a new era of prognostic and therapeutics targets in next generation of cancer management [7]. Interestingly, bone marrow derived endothelial progenitor cells shown to act as critical regulators of angiogenic switch and that ultimately regulates pulmonary metastasis of cancer cells and further indicated that tumor-stromal interaction played crucial role in tumor metastasis and angiogenesis [8]. Moreover, using an activity based protein profiling approach; Jessani *et al.* revealed the elevated enzymatic activity of serine proteases uPA and tPA of human breast cancer cells in host environment of mouse mammary fat pad that regulates breast cancer progression [9].

OPN plays crucial role in various physiological as well as pathological functions [10]–[14]. OPN activates multiple signaling cascades that regulates the expression of various oncogenic and angiogenic molecules ultimately leading to tumor progression [10], [14], [15]. Highly malignant tumors express enhanced OPN expression as compared to benign ones [16]–[19]. Furthermore, targeted disruption or inhibition of tumor derived OPN significantly curbs tumor progression, metastasis and angiogenesis in *vitro* as well as *in vivo* [20]–[28]. Recently, Grassinger *et al.* have demonstrated that thrombin cleaved OPN acts as a chemoattractant for stem and progenitor cells [29]. Moreover, OPN is a key component of hematopoietic stem cell niche that negatively regulates stem cell pool size and controls primitive hematopoietic progenitor cells [30], [31]. However, Sumitomo *et al.* have observed that transcriptional mediator subunit MED1/TRAP220 in stromal cells promotes hematopoietic stem/progenitor cell growth through OPN expression [32]. Likewise, Saika *et al.* have demonstrated that loss of OPN in an injured mouse lens epithelium perturbs the epithelial-mesenchymal transition suggesting the importance of OPN in EMT [33]. Recently, it has been shown that OPN derived from senescent fibroblast stimulates preneoplastic cell growth through CD44 receptor and MAPK activation pathway, highlighting the importance of stromal OPN on tumorigenesis [34], [35]. Moreover, adding exogenous OPN to breast cancer cells promotes cell survival, angiogenesis and tumor growth [20], [22]. Furthermore, tissue microarrays and individual skin biopsies of different stages of melanoma revealed that enhanced OPN expression correlates with melanoma invasion [36]. In addition, gene expression data confirms that OPN may act as prognostic marker in melanoma [37]. However, the role of stroma/host-derived OPN in regulation of melanoma progression is not clearly understood and is the subject of intense investigation.

In the present study, we provide both *in vitro* and *in vivo* experimental evidences that host OPN regulate melanoma growth, angiogenesis and metastasis. Moreover, we have shown that melanoma cells derived from OPN^+/+^ mice exhibit enhanced tumorigenic feature as compared to parental or cell derived from OPN^−/−^ mice. The data revealed that stromal OPN fostering stem like cancer cell growth and regulate melanoma angiogenesis and metastasis. Furthermore, we have demonstrated at least in part, the molecular mechanism by which stromal OPN regulates ERK signaling and ABCG2 expression leading to enrichment of stem like B16F10 cells. Thus, our finding suggests that stroma-derived OPN regulates melanoma growth and may act as prognostic marker and therapeutic target in melanoma microenvironment.

## Materials and Methods

### Antibodies and other Reagents

Rabbit anti-ABCG2, anti-phospho-Akt and anti-ERK; mouse anti-phospho-ERK and goat anti-Akt2 and anti-actin were purchased from Santa Cruz Biotechnology (Santa Cruz, CA). Goat anti-OPN, MTT (3-(4,5-dimethylthiazol-2-yl)-2,5-diphenyl tetrazolium bromide), Hoechst 33342, reserpine, wortmannin and U0126 were procured from Sigma (St. Louis, MO). Boyden type cell migration chamber was obtained from Corning (Corning, NY). Matrigel and Matrigel coated invasion chamber were obtained from BD Bioscience (Bedford, MA). Wild type C57 (OPN^+/+^) and OPN knockout (OPN^−/−^) (C57BL/6Jx129/SvJ, Strain name: B6.Cg-Spp1tm1Blh/J) mice were obtained from Jackson Laboratory (Bar Harbor, ME), and maintained at NCCS Experimental Animal Facility (EAF) with approved ethical guidelines.

### Mammalian Cell Culture

The murine melanoma cell line B16F10 were purchased from American Type Culture Collection (ATCC, Manassas VA) and cultured in RPMI supplemented with 10% fetal bovine serum, 100 units/ml penicillin and 100 mg/ml streptomycin in a humidified atmosphere of 5% CO_2_ and 95% air at 37°C. Human umbilical vein endothelial cells (HUVEC) were obtained from Lonza (Walkersville, MD) and cultured as per manufacturer’s instructions. B16F10-Luc cells were procured from Xenogen Corporation (Alameda, CA).

### Development of Melanoma Growth and Establishment of Primary Melanoma Cells

All procedures involving mice and experimental protocols were approved by Institutional Animal Care and Use Committee (IACUC) of National Centre for Cell Science, Pune, India. B16F10 (2×10^5^) cells were injected subcutaneously into the dorsal flank region of OPN^+/+^ and OPN ^−/−^ mice (6–8 weeks old). Tumor growth was measured every week. Mice were sacrificed after 5 weeks, photographed; tumors were dissected out, weighed and used for further studies.

For primary culture, tumor bearing mice from each group were selected randomly, sacrificed by cervical dislocation, tumors were isolated and rinsed twice in sterile PBS. Tumors were minced and digested in trypsin-EDTA (0.5% trypsin and 50 mM EDTA) for 30 min. Cells collected were washed with PBS, supplemented with complete media and incubated in humidified atmosphere of 5% CO_2_ and 95% air at 37°C. The cells derived from OPN^+/+^ and OPN^−/−^ mice denoted as B16-WT and B16-KO respectively. These cells were used maximum to 4 passages for characterization and further mechanistic studies.

### *In vivo* Metastasis Study by Intra-cardiac and Intra-venous (Trough Tail Vein) Injection

For metastasis studies, B16F10 cells (1×10^5^) were injected into the left ventricle of anaesthetized OPN^+/+^ and OPN ^−/−^ female mice (6–8 weeks old). After 21 days, mice were sacrificed; photographed and internal organs such as lung and liver were dissected out for histopathological studies. In separate experiments, B16F10, B16-WT or B16-KO cells (1×10^5^) were injected at intra-cardiac position of anaesthetized OPN^−/−^ female mice. After 21 days, mice were sacrificed and metastasis of melanoma was analyzed as described above.

In another experiments, sorted SP and non SP (NSP) from B16F10-Luc cells (1×10^3^) were injected into the lateral tail vein of NOD/SCID mice (6–8 weeks old). After 21 days, mice were sacrificed; lung and liver were removed and analyzed for distant metastasis using *In Vivo* Imaging System (IVIS).

### Hoechst 33342 Staining and Side Population (SP) Analysis

Flow cytometry with Hoechst 33342 was performed as described earlier with minor modifications [38]. Briefly, cells (1×10^6^/ml) were resuspended in Hank’s balanced salt solution (HBSS) containing 2% fetal bovine serum (FBS) and 25 mM HEPES. Cells were preincubated with 50 µM reserpine at 37°C for 15 min to inhibit ABC transporters and further incubated with 5 µg/ml of Hoechst 33342 for 90 min at 37°C. Cells were washed with ice-cold HBSS and analyzed using Hoechst 33342 at excitation of 407 nm by Trigon violet laser and dual wavelengths of 450/40 (Hoechst 33342-Blue) and 695/40 (Hoechst 33342-Red) filters on FACSAria (BD Biosciences, San Jose, CA). Dead cells were excluded by gating on forward and side scatter and PI-positive population was eliminated. Hoechst red channel is more sensitive and therefore small change in dye concentration results in distinct tail of cells and often referred as ‘Side Population’.

### Western Blot Analysis

To determine the expression and phosphorylation of various downstream signaling molecules those of which are regulated in response to stroma-derived OPN, western blot was performed from the cell lysates of parental B16F10, B16-WT or B16-KO cells as described earlier [39]. Briefly, these cells were lysed in lysis buffer, total proteins (40 µg) were resolved by SDS-PAGE and blotted onto nitrocellulose membranes. The levels of OPN, VEGF, phosphor-ERK1/2, ERK2, phospho-Akt1/2, Akt2, ABCG2 and actin were analyzed by western blot using their specific antibodies. In separate experiments, B16F10 cells were treated with conditioned media (CM) collected from B16-KO or B16-WT cells. In another experiments, CM of B16WT treated cells were incubated with wortmannin or U0126 and the level of ABCG2 was analyzed. In other experiments, B16F10 cells were stably transfected with ERK1-wt, ERK1-dn, ERK2-wt or ERK2-dn construct and the lysates were analyzed for ABCG2 by western blot.

### Matrigel Tube Formation Assay

To determine the vasculogenic mimicry of melanoma cells, tube formation assay was performed as described [40]. Briefly, 50 µl of growth factor depleted matrigel was coated on 96 well plates for polymerization and melanoma (B16F10, B16-WT and B16-KO) cells (1×10^4^ cells/well) were added. After 8 h, photographs were captured using inverted microscope (Nikon), and the tubular alignment formed were analyzed statistically and represented as mean±SE. All the experiments were performed in triplicates.

### Wound Migration Assay

To determine the motility of parental B16F10, B16-WT or B16-KO cells, wound assay was performed [40]. Briefly, B16F10, B16-KO or B16-WT cells were grown in monolayer and synchronized in serum depleted medium. Wound with uniform size was made using sterile tip and photographed at t = 0 h and t = 12 h using phase contrast microscope (Nikon). Wound closure was measured by Image-Pro plus software, analyzed statistically and represented in the form of bar graph.

### *In vitro* Clonogenicity Assay

*In vitro* clonogenicity assay was performed as described earlier [40]. Briefly, 50 µl of growth factor depleted matrigel was coated in 96 well plate and equal numbers of B16F10, B16-KO and B16-WT cells were added to it and incubated at 37°C. In separate experiments, equal numbers of sorted SP or non-SP cells were plated on matrigel coated plate. After 10 days, colonies were visualized under microscope (Nikon), photographed, counted, analyzed statistically and represented graphically.

### Migration, Invasion, Co-migration and Co-invasion Assays

Migration or invasion assays were performed in modified Boyden chamber or matrigel coated invasion chamber as described earlier [22], [40]. The endothelial-tumor cell interaction was shown by direct co-migration or co-invasion assays. HUVECs were plated in upper chamber and B16F10, B16-WT or B16-KO were plated in lower chamber of modified Boyden chamber or matrigel coated invasion chamber and the co-migration and co-invasion assays were performed. After 12 h, the migrated or invaded HUVEC in the reverse side of the filter were stained with Giemsa, photographed, analyzed as described above and represented graphically.

### Statistical Analysis

The tumor volumes were measured and represented in the form of line graph. The migration, invasion, tumor-endothelial interaction assays, tumor weight and angiogenesis assay were analyzed statistically and represented in the form of bar graph using Sigma Plot. Statistical differences were determined by Student’s t test. Differences were considered significant when the *P* value was less than 0.05.

## Results

### Abrogation of Melanoma Growth and Angiogenesis in OPN^−/−^ Mice

**Figure 1:**
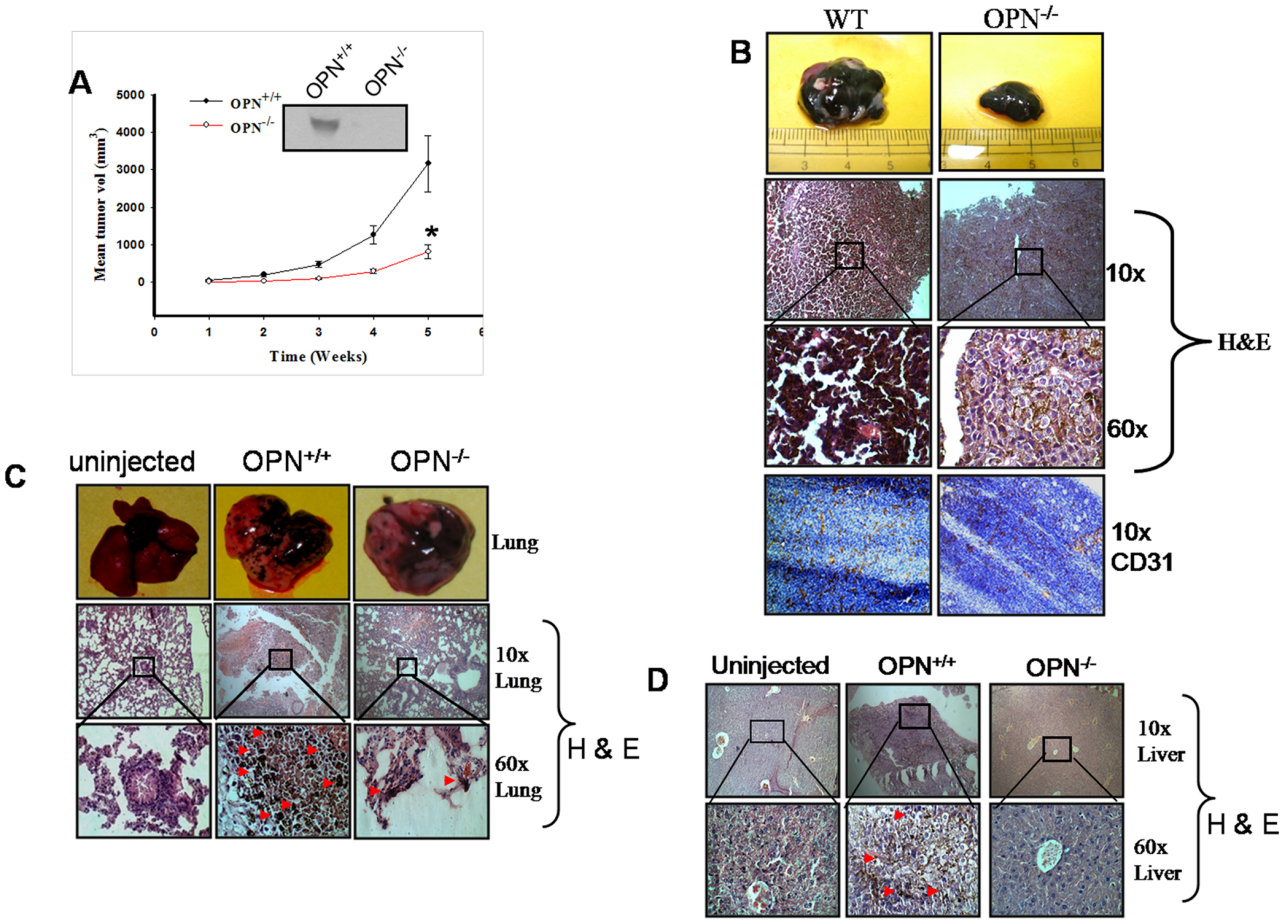
Attenuation of melanoma growth, angiogenesis and metastasis in OPN^−/−^ mice. (**A**) B16F10 cells were injected subcutaneously into OPN^+/+^ and OPN^−/−^ mice (n = 6 mice per group). Tumor growth kinetics in OPN^+/+^ and OPN^−/−^ mice were measured weekly and represented graphically. Error bar, SEM (*, P = 0.013). *Inset:* western blot analysis for OPN expression in serum of OPN^+/+^ and OPN^−/−^ mice. (**B**) Mice were sacrificed, tumors dissected out, photographed, weighed and analyzed histopathologically. *Upper panel*: representative image of tumor from the cohorts of OPN^+/+^ and OPN^−/−^ mice. *Middle panel*: tumors dissected were sectioned and stained with hematoxylin and eosin, and analyzed histopathologically. Images were captured at 10× and 60×magnifications. *Lower panel*: Tumor angiogenesis were studied from the cryo-sectioned tumor tissue using anti-CD31 antibody. CD31 are stained with DAB (brown) whereas nuclei are stained with hematoxylin (blue). Photographs were captured at 10×magnification. (**C**) B16F10 cells were injected intracardiacly to OPN^+/+^ and OPN^−/−^ mice. Mice were dissected; lung tissue was collected and photographed after experimental metastasis assay. *Upper panel*: Lung photographs of representative experimental mice group. Uninjected mice were used as control. *Middle and lower panels*: histopathological analysis of lung sections to study metastasis. Images were captured at 10× and 60×magnifications. (**D**) Analysis of liver metastasis was performed histopathologically, and images were captured at 10× and 60×magnifications. Metastatic foci in liver sections were indicated by arrow.

The role of tumor derived OPN in regulation of tumor progression is well established, however, role of host derived OPN in tumorigenesis is not clearly understood. Recently, Chakraborty *et al.* have shown that abrogation of host OPN significantly suppressed *in vivo* breast tumor growth in mice model [41]. Earlier, Hayashi *et al.* reported that serum collected from OPN^+/+^ mice induced *in vitro* migration of B16F10 cells whereas serum from OPN^−/−^ mice suppressed this event which further suggested the potential role of host OPN in tumor progression [42]. Therefore, to explore the role of stroma derived OPN in melanoma growth, B16F10 cells were injected into the OPN^+/+^ and OPN^−/−^ mice. Tumors growth was measured weekly and plotted graphically. The data illustrated that deficiency of host OPN significantly reduced *in vivo* melanoma growth (Fig. 1A). Serum was collected from these mice and OPN expression was analyzed by western blot. The data showed significant OPN expression in the sera of OPN^+/+^ whereas it was absent in sera of OPN^−/−^ mice (Fig. 1A: *inset*). Tumor weights were measured and represented graphically, and the data reflects about 4 fold reduction of melanoma load that occurs due to deficiency of host OPN (Fig. S1A). Histopathological analysis (H&E staining) of tumor sections showed higher infiltration and poorly differentiated structure in OPN^+/+^ mice as compared to OPN^−/−^ (Fig. 1B, middle panel). To determine whether host OPN promote melanoma angiogenesis, tumor cryosections were stained with anti-CD31 antibody and the results showed higher angiogenesis (CD31 positive areas) in the tumor derived from OPN^+/+^ as compared to tumors of OPN^−/−^ mice (Fig. 1B, lower panel). Taken together these data suggested that host OPN is crucial for melanoma growth and angiogenesis *in vivo*.

### Stromal OPN Promotes Lung and Liver Metastases

Several reports suggest that tumor derived OPN augments metastasis in various cancer models by inducing the expression of varieties of oncogenic molecules through multiple signaling cascades [14]. However; it is not clear whether host OPN has any role in melanoma metastasis. Thus, to assess the role of host OPN in experimental melanoma metastasis, B16F10 cells were injected to OPN^+/+^ and OPN^−/−^ mice intracardiacly. After termination of experiments, mice were sacrificed and lungs and liver were dissected and photographed. Enhanced melanoma metastases were observed in the lung of OPN^+/+^ as compared to OPN^−/−^ mice (Fig. 1C, upper panel).

The lung and liver sections were analyzed by H&E staining and significant number of metastatic foci were visible in the lung of OPN^+/+^ mice as compared to OPN^−/−^ mice (Fig. 1C). Moreover, metastatic foci of melanoma were observed in liver of OPN^+/+^ but not in OPN^−/−^ mice as indicated by arrow (Fig. 1D).

### Functional Characterization of Melanoma Cells Isolated from OPN^+/+^ and OPN^−/−^ Mice Tumors

**Figure 2:**
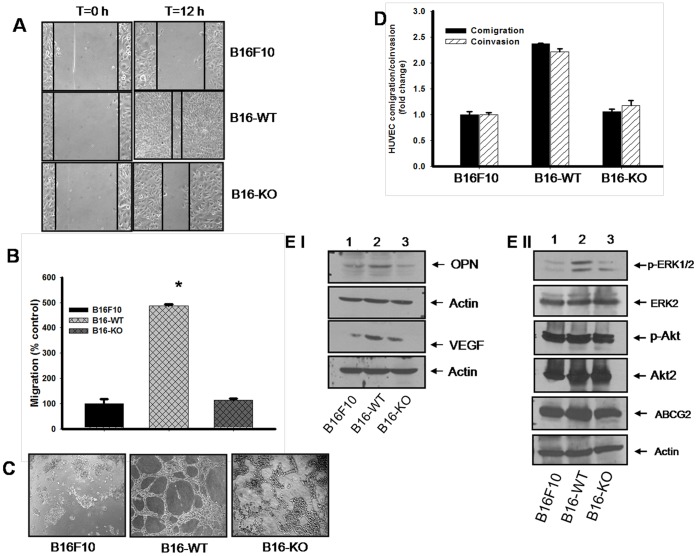
Characterization of primary tumor cells derived from B16F10 generated tumors of OPN^+/+^ and OPN^−/−^ mice. (**A**) Artificial wounds were made in confluent monolayer of B16F10, B16-WT and B16-KO cells. Migration of melanoma cells towards the wound were photographed after 12 h. (**B**) Melanoma cells (B16F10 or B16-WTor B16-KO) were plated on the upper chamber of transwell whereas lower chamber were filled with medium containing 2% FBS. Migrated cells in the opposite side of upper chamber were stained with Giemsa and photographed, counted, analyzed statistically and represented in the form of bar graph. Error bar depicts mean±SEM. *, P<0.001 vs. control. (**C**) Parental as well as primary cultures were plated on the matrigel coated plate for 8 h. Tubes formed were photographed. (**D**) Melanoma cells were seeded in the lower chamber whereas HUVEC (1×10^5^) were plated in the upper chamber of modified Boyden chamber or matrigel coated invasion chamber and incubated for 12 h. Migrated or invaded HUVEC on the opposite side of upper chamber were stained with Giemsa and photographed. Migrated and invaded endothelial cells were counted, analyzed statistically and represented graphically. Graph, representative of three identical repeats depict mean±SEM. *, P<0.001 vs. control. (**E**) Western blots analyses of OPN, VEGF (panel I), p-ERK, p-Akt and ABCG2 (panel II) in B16F10, B16-WT and B16-KO cell lysates. Actin, Akt2 and ERK2 were used as loading control. All the panels are representation of three independent experiment showing similar results.

To explore the role of host derived OPN in regulation of metastatic and aggressive ability of melanoma, cells were isolated from subcutaneous melanoma tumors of OPN^+/+^ and OPN^−/−^ mice and cultured under *in vitro* conditions. Cells isolated from OPN^+/+^ and OPN^−/−^ mice were termed as B16-WT and B16-KO respectively; and used throughout the study. Morphologically B16-WT and B16-KO cells do not show any significant differences from parental B16F10 cells (Fig. S1B). However, B16-WT cells exhibited higher migratory activity under *in vitro* wound assay than parental B16F10 or B16-KO cells (Fig. 2A and Fig. S1C). To further confirm this observation, transwell migration assay was performed using Boyden chamber and the results revealed higher B16-WT cell migration as compared to parental B16F10 or B16-KO cells (Fig. S1D and Fig. 2B). These data demonstrated that B16-WT cells acquired enhanced migratory activity than B16F10 or B16-KO cells.

### B16-WT Cells Exhibit Vasculogenic Mimicry and Tumor-endothelial Cell Interaction

Vasculogenic mimicry is correlated with poor prognosis and enables melanoma growth, at least in part, independent of angiogenesis [43]. Previous studies have suggested that highly aggressive melanoma cells form ECM-rich tubular network that can ensure blood and nutrient supply to tumors and this phenomena is termed as vasculogenic mimicry [44]. Recently, Rothhammer *et al* have shown that highly aggressive melanoma cells efficiently formed capillary like network on matrigel coated plate and indicated that these cells have the potential to exhibit vasculogenic mimicry [45]. Therefore, to assess whether enhanced aggressiveness of B16-WT cells reflects the phenomenon of vasculogenic mimicry; cells (B16F10, B16-WT and B16-KO) were plated on matrigel coated plate and incubated further. The results revealed that B16-WT cells formed tube like network within 8 h (Fig. 2C & Fig. S1E). No significant tube-like network formation was found in parental B16F10 or B16-KO cells even after 24 h.

During metastasis, tumor cells interact with the nearby endothelial cells of blood vessels, invade and metastasize through the blood stream to distant organs. Therefore, to assess the direct interaction between melanoma and endothelial cells, co-migration and co-invasion assay was performed. The migrated or invaded HUVEC on the reverse side of upper chamber were stained with Giemsa, photographed in three hpf (high power field), counted, analyzed and represented in the form of bar graph (Fig. S2A and B and Fig. 2D). The data depicted that B16-WT cells promote endothelial cells migration/invasion and further suggested that these cells exhibit enhanced angiogenic potential as compared to B16F10 and B16-KO cells. Taken together these data suggested that melanoma cells isolated for wild type mice exhibit enhanced aggressive invasive and migratory behavior as compared to parental or melanoma cell derived from OPN^−/−^.

### Expression and Activation of Signaling Molecules in Parental and Primary Melanoma Cells

B16-WT cells exhibited higher oncogenic as well as angiogenic behavior as compared to parental B16F10 or B16-KO cells. Therefore, we hypothesized that host derived OPN might induce constitutive alteration of expression profile of various oncogenic molecules which in-turn regulates the aggressive behavior of B16-WT cells. Accordingly, western blot analysis from the lysates of B16-WT, B16-KO and B16F10 were performed to check the level of OPN and VEGF. The result revealed that B16-WT cells exhibit higher levels of OPN and VEGF expression as compared to B16F10 and B16-KO cells (Fig. 2E, panel I). We further examined whether there are any differential regulation of signaling events occurred in these cells. Accordingly, expressions of ABCG2, p-Akt and p-ERK were detected by western blot analysis. The results showed that expressions of ABCG2 and p-ERK are augmented in B16-WT cells as compared to B16F10 or B16-KO cells suggesting the importance of stromal OPN in regulation of this process (Fig. 2E, panel II). Interestingly, no significant difference of p-Akt expression was observed in these cells.

### Reintroduction of B16-WT Cells in OPN^−/−^ Mice Exhibit Enhanced Tumor Growth and Metastasis

**Figure 3:**
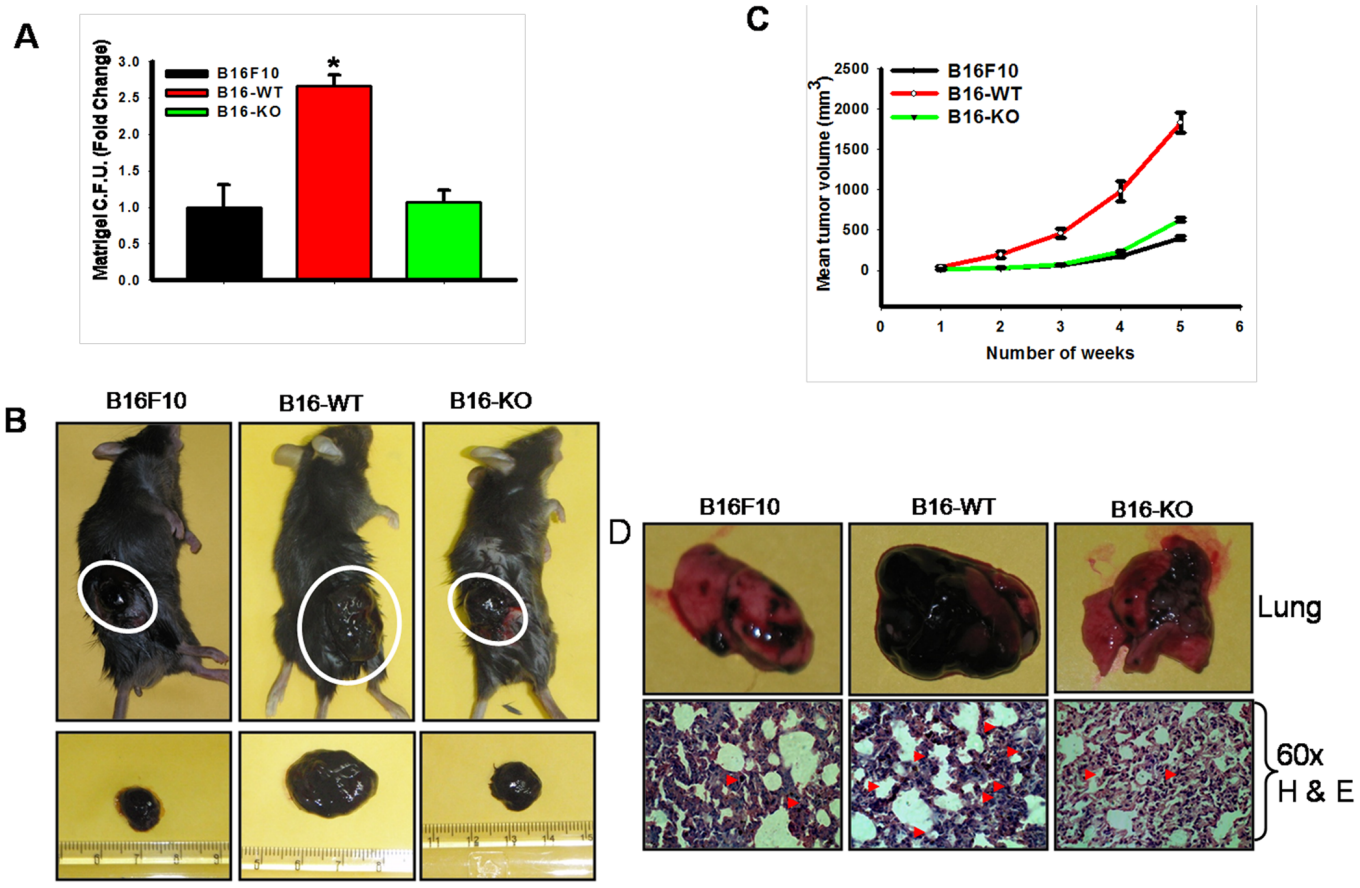
B16-WT cells showed enhanced tumorigenic, angiogenic and metastatic property in experimental mice model. (**A**) B16F10, B16-WT and B16-KO cells were plated onto matrigel coated plate and allowed to grow for 10 days. Media were replaced every alternate day. Colonies formed were photographed, counted, analyzed statistically and represented graphically. Bars, mean±SEM (*, P<0.021 vs. control). The data shown is the representation of three independent experiment showing similar results. (**B**) B16F10, B16-WT and B16-KO cells were injected subcutaneously to the OPN^−/−^ mice. After termination of experiments (5 weeks), mice were sacrificed and tumors were isolated and photographed. (**C**) Tumor volumes were measured weekly (until 5 weeks), analyzed statistically and represented graphically. *, P<0.001 vs. control. (**D**) B16F10, B16-WT and B16-KO cells were injected at intracardiac position of OPN^−/−^ mice to study lung metastasis. After 21 day, mice were sacrificed and lung was dissected out. *Upper panel*: Photographs of representative lung isolated from experimental mice. Lower panel: Lungs were analyzed histopathologically and photographs were taken at 60× magnification.

To study the *in vitro* tumorigenic potential of B16-WT cells, matrigel based colony formation assay was performed. Interestingly, these cells showed considerably higher *in vitro* colony formation with respect to B16-KO or parental B16F10 cells (Fig. 3A). This observation prompted us to determine the *in vivo* tumor forming capacity of B16-WT or B16-KO cells. Accordingly, cells (B16F10, B16-WT or B16-KO) were implanted subcutaneously onto the OPN^−/−^ mice. Tumor growths were measured weekly up to five weeks. Mice were sacrificed and tumors were photographed (Fig. 3B). Tumor volume and weight were analyzed and represented in the form of graph (Fig. 3C & Fig. S2C). The data showed that B16-WT cells exhibit significantly higher tumor load as compared to B16-KO or parental B16F10 cells.

To examine the comparative metastatic potential of B16-WT and B16-KO cells, *in vivo* metastasis assay was performed. Cells were injected to OPN^−/−^ mice through intracardiac route and kept for 21 days. After termination of experiments, mice were sacrificed, lungs were removed and analyzed histopathologically and the result showed comparatively higher lung metastasis in B16-WT cells when injected to OPN^−/−^ mice (Fig. 3D). Taken together, these data demonstrated that B16-WT cells exhibit enhanced melanoma growth and metastasis suggesting the importance of stromal OPN in this process.

### Stromal OPN Selectively Enriches SP Phenotype in Murine Melanoma Cells

**Figure 4:**
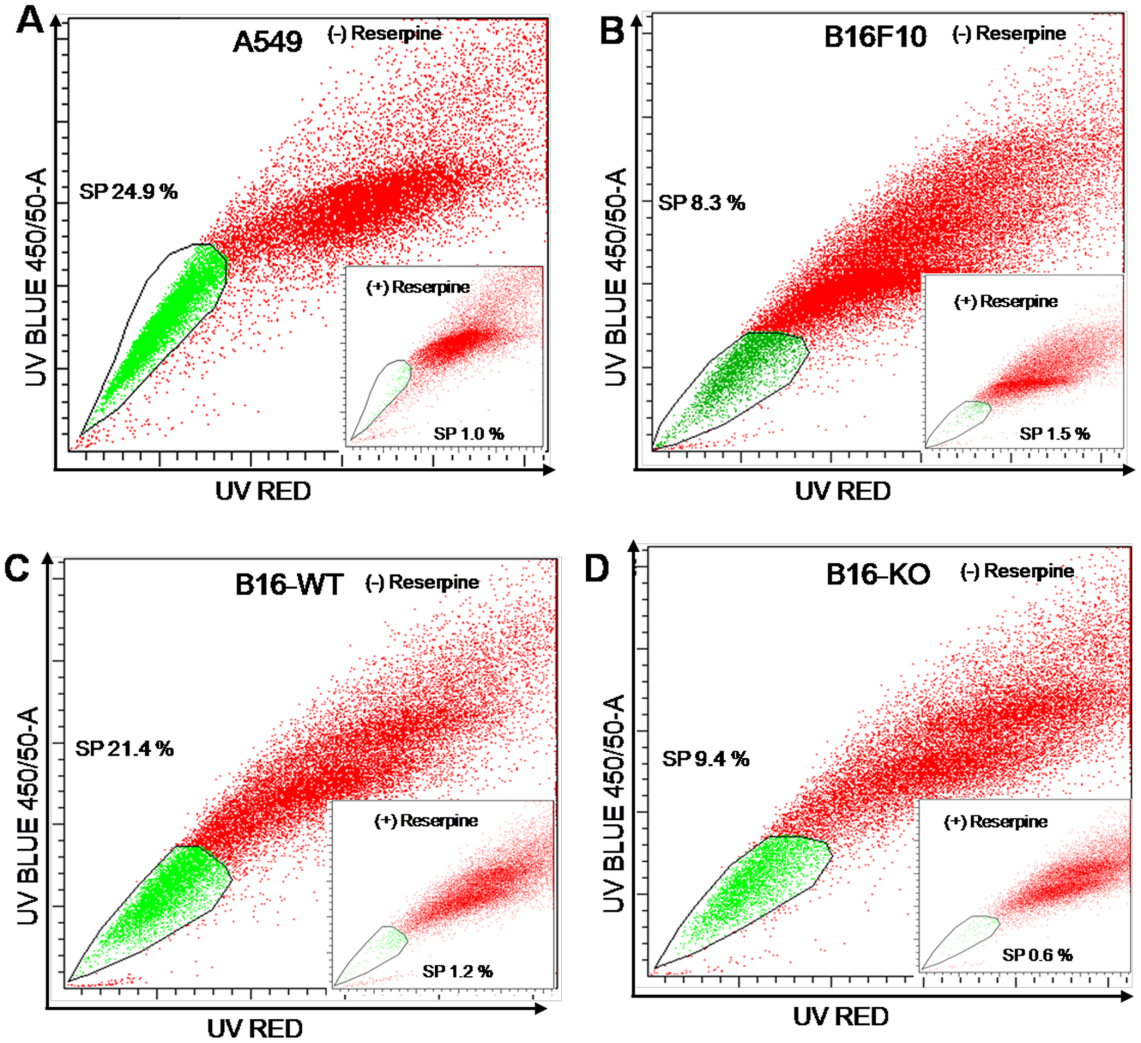
Host OPN selectively enriches stem-like cancer cells in B16F10 cells. (**A-D**) Cells (B16F10, B16-WT, B16-KO) were stained with Hoechst 33342 dyes in the absence or presence of reserpine and analyzed using flow cytometer for SP population. Lung adenocarcinoma (A549) cells used as positive control for SP phenotype. The average size of the SP was around 8–9% in B16F10 and B16-KO cells whereas 21% in B16-WT cells. All data are representation of three independent experiments exhibiting similar results.

Side population (SP) is a set of cells with differential exclusion of Hoechst dye through ABC transporter and expected to exhibit the property of cancer stem cells. Recently, Dou *et al* have reported the presence of SP phenotype in B16F10 melanoma cells [46]. However, the impact of stromal-OPN on regulation of melanoma cancer stem cell or tumor initiating cells has not been studied so far. Therefore, to study this effect, cells (B16F10, B16-WT and B16-KO) were stained with Hoechst and analyzed by flow cytometry. To confirm the existence of SP, reserpine is used in each experimental condition. Lung adenocarcinoma (A549) cells were used as positive control for SP phenotype. The percentage of SP phenotype observed was 24.9%, 8.3%, 21.4% and 9.4% in A549, B16F10, B16-WT and B16-KO cells respectively (Fig. 4A–D). The data clearly demonstrated that stromal OPN selectively enriches SP phenotype.

Several reports have suggested that host cell particularly endothelial or fibroblast cells exhibit SP phenotype. Accordingly, to over rule that increase in SP phenotype is not due to accompanying host cells, B16F10 cells were stably transfected with GFP expression vector (pEGFP N1) and injected into OPN^+/+^ and OPN^−/−^ mice. Primary cultures from these tumors (B16-WT-GFP and B16-KO-GFP) were established and used for SP analysis. SP analysis was performed for GFP positive WT and KO cells. The results demonstrated that higher percentage of SP phenotype were observed in B16-WT-GFP as compared to B16-KO-GFP suggesting that this enrichment of SP cells derived from tumor is due to stromal OPN (Fig. S3A–F & Fig. S4A–F).

### Efflux of Mitoxantrone from B16F10 SP Cells

**Figure 5:**
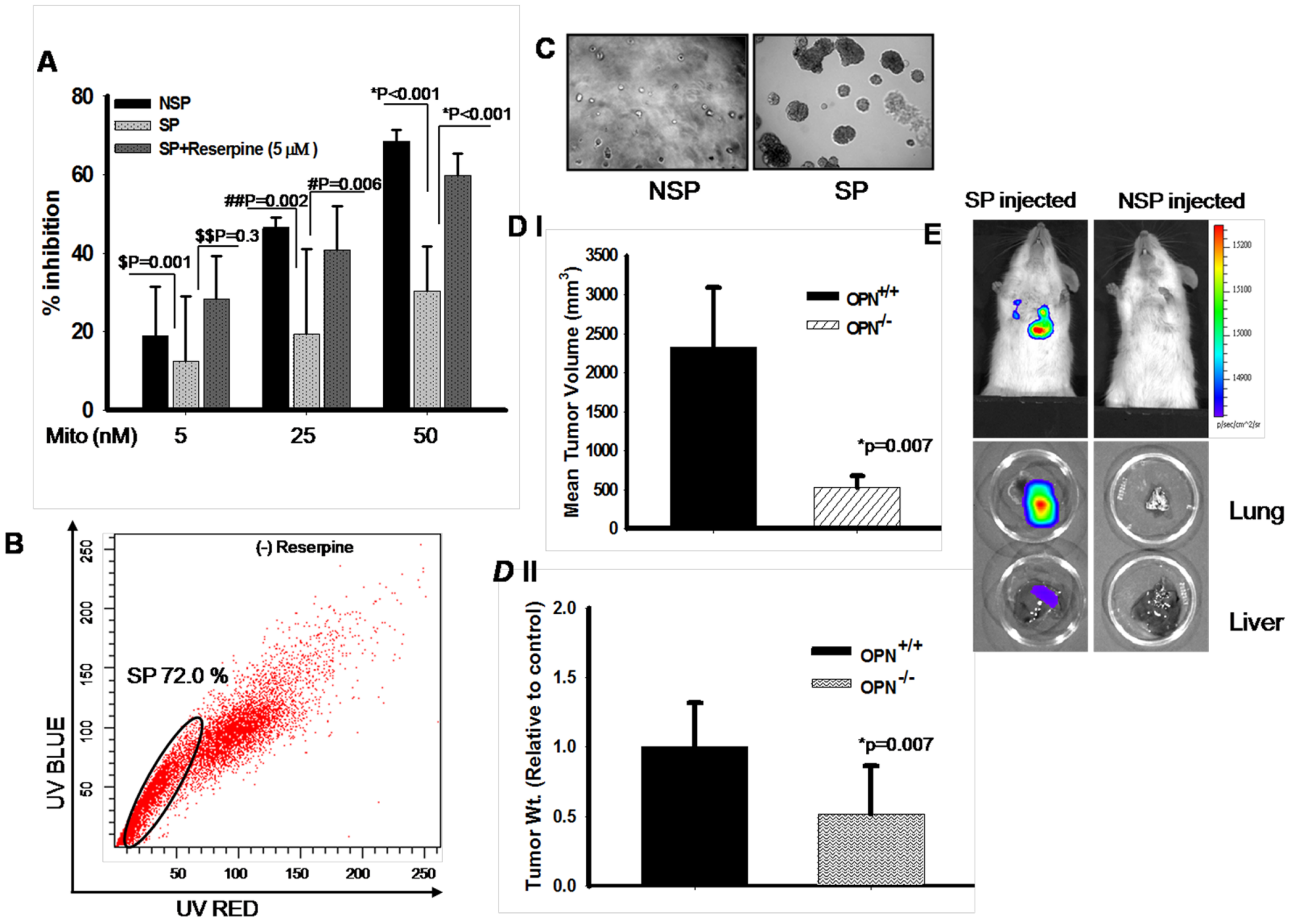
Sorting and characterization of SP and non-SP from B16F10 cells. (**A**) Sorted SP and non-SP cells were treated with mitoxantrone (0–50 nM). In addition, SP cells were treated with or without reserpine (5 µM) and growth inhibition analysis was performed by MTT assay. Results are mean ± SEM (^$^, P = 0.001; ^$$^, P = 0.3; ^#^, P = 0.006; ^##^, P = 0.002; *, P<0.001). The data shown is the representation of three independent experiments showing similar results. (**B**) Differentiation assay was performed with *in vitro* cultured SP cells. The data are the representation of three independent experiments showing similar results. (**C**) *In vitro* tumorigenicity of SP and NSP cells were checked by Matrigel-based colony formation assay. Colonies formed were photographed at 10× magnification. The data shown is representation of three separate experiments showing similar results. (**D**) (*panel I*), Sorted SP cells (1×10^3^) were injected into the OPN^+/+^ and OPN^−/−^ and kept for 5 weeks. Mice were sacrificed and tumors were collected and photographed. Mean tumor volume was calculated, analyzed statistically and represented graphically. Bar represents mean±SEM; *, P = 0.007. *Panel II*, Mean tumor weight was calculated, analyzed statistically and represented in the form of bar graph. Bar represents mean±SD; *P = 0.007. (**E**) Sorted SP and non-SP cells were injected intravenously into NOD/SCID mice (n = 9) and metastasis to lungs and liver was analyzed using IVIS system. *Lower panel*: Mice were sacrificed. Lung and liver were dissected out and metastases were analyzed using IVIS.

The enhanced expression of ABC transporter proteins indicates that the SP cells should have high efflux capacity for anti-neoplastic drugs. To investigate this possibility, SP cells were sorted and treated with mitoxantrone in absence or presence of reserpine for 24h. Similarly, non-SP cells were also treated with mitoxantrone and cell viability assay was performed. The results showed that the increase in inhibition of cell growth was observed in non-SP cells as compared to SP cells with increasing doses of mitoxantrone. However, reserpine treated SP cells restored the growth inhibition in presence of mitoxantrone (Fig. 5A). Taken together, these data suggested that mitoxantrone resistance is due to its efflux by the ABC-transporter, and was blocked upon reserpine treatment.

### SP Regenerates into SP and non-SP Cells

To compare the differentiation ability of SP cells, we cultured the sorted SP cells and then stained with Hoechst 33342 dyes and analyzed by flow cytometry. SP cells were viable in culture and regenerated to both SP and non-SP cells with a higher percentage of SP (72%) (Fig. 5B). These data suggested that SP cells have the property of differentiation which is a typical feature of stem or progenitor cells.

### SP Cells are more Tumorigenic and Metastatic in Nature

To further examine whether SP cells are more tumorigenic, *in vitro* colony formation assay was performed with sorted SP and non-SP cells. The data revealed that SP cells have higher colony formation ability than non-SP cells (Fig. 5C & Fig. S2D). To further examine, sorted SP and non-SP cells (1×10^3^) were injected subcutaneously into OPN^+/+^ and OPN^−/−^ mice and allowed to grow the tumor for 5 weeks. Tumor generated in OPN^+/+^ and OPN^−/−^ mice were sacrificed, tumors were dissected, weighed and tumor volume was calculated. Enhanced tumor growth was observed when SP cells were injected in OPN^+/+^ as compared to OPN^−/−^ mice (Fig. S2E and Fig. 5D, panels I & II). Non-SP cells showed significantly less tumorigenic potential as compared to SP cells (Table 1).

**Table 1:** Summary of tumor incidence in OPN^+/+^ and OPN^−/−^ mice upon injecting with SP and non-SP cells.

Injected (1×10^3^ cells/mice)	Tumor incidence in OPN^+/+^ mice	Tumor incidence in OPN^−/−^ mice
SP	10/12	7/12
NSP	2/12	0/12

To investigate possible differences in metastasis between SP and non-SP, sorted cells (SP and NSP) obtained from B16F10-Luc were injected into NOD/SCID mice through *i.v*. and kept for 21 days. *In Vivo* Imaging System (IVIS) analyses were performed, mice were sacrificed; lung and liver were dissected out and their images were taken further (Fig. 5E). The data demonstrated the enhanced metastatic ability of SP cells into lung and liver than non-SP cells.

### Stromal OPN Regulates SP Phenotype through Modulating ERK Signaling

**Figure 6:**
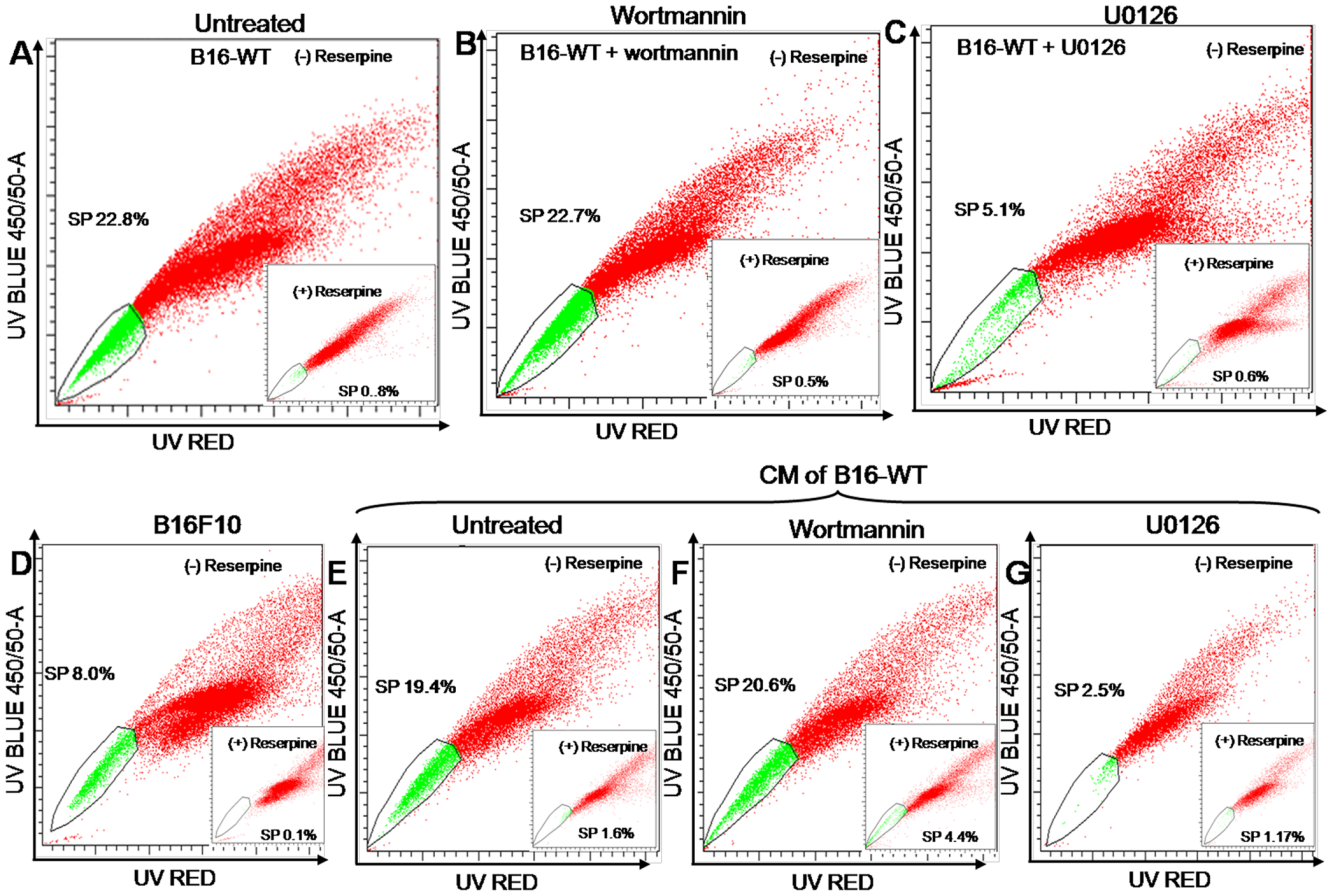
Stromal OPN regulates SP phenotype in B16F10 through ERK pathway. (***A***) B16-WT cells were stained with Hoechst and SP analysis was performed with flow cytometer. (***B***) B16-WT cells were treated with wortmannin for 24 h, stained with Hoechst and analyzed for SP phenotype. (***C***) B16-WT cells were treated with U0126 and SP analysis was performed. (***D***) Flow cytometeric analysis of SP phenotype in B16F10 cells. (***E***) B16F10 cells were treated with conditioned media of B16-WT cells for 24 h and SP analysis was performed. (***F***) B16F10 cells were pre-treated with conditioned media of B16-WT cells and then treated with wortmannin for 24 h and SP analysis were performed. (***G***) B16F10 cells were pre-treated with conditioned media of B16-WT cells and then treated with U0126 for 24 h and SP analysis was performed. ***Inset:*** control setup for SP analysis treated with reserpine for respective panels. All the panels are representative of three independent repeats exhibiting similar results.

To examine the signaling pathway involved in enrichment of SP phenotype, B16-WT cells were either treated with PI3 kinase inhibitor (wortmannin) or MEK/ERK inhibitor (U0126) for 24 h, stained with Hoechst and analyzed by flow cytometry. The results showed that blocking PI3-kinase have no effect on SP pool size, however when treated with U0126, SP phenotype decreased significantly (Fig. 6A–C), suggesting that ERK pathway is involved in the regulation of enhanced SP phenotype. To examine whether B16-WT cells has the ability to enrich SP phenotype in parental B16F10 cells, conditioned media (CM) collected from B16-WT cells were used to treat B16F10 cells, stained with Hoechst dyes and analyzed by flow cytometer. The results depicted almost two fold increase in SP phenotype in CM treated B16F10 cells (from 8% to 19.4%) as compared to untreated parental B16F10 cells (Fig. 6D & E). We further treated B16F10 cells either with wortmannin or U0126 along with CM of B16-WT for 24 h, stained with Hoechst dyes and SP analysis was performed. The results showed that blocking PI3K signaling has no effect on SP pool size of CM treated B16F10 cells (Fig. 6F) whereas inhibiting MAPK pathway by U0126 significantly reduces the SP phenotype to 2.5% in CM treated B16F10 cells (Fig. 6G). This observation further supported the earlier findings and suggested that stromal-OPN regulates SP phenotype through ERK pathway.

**Figure 7:**
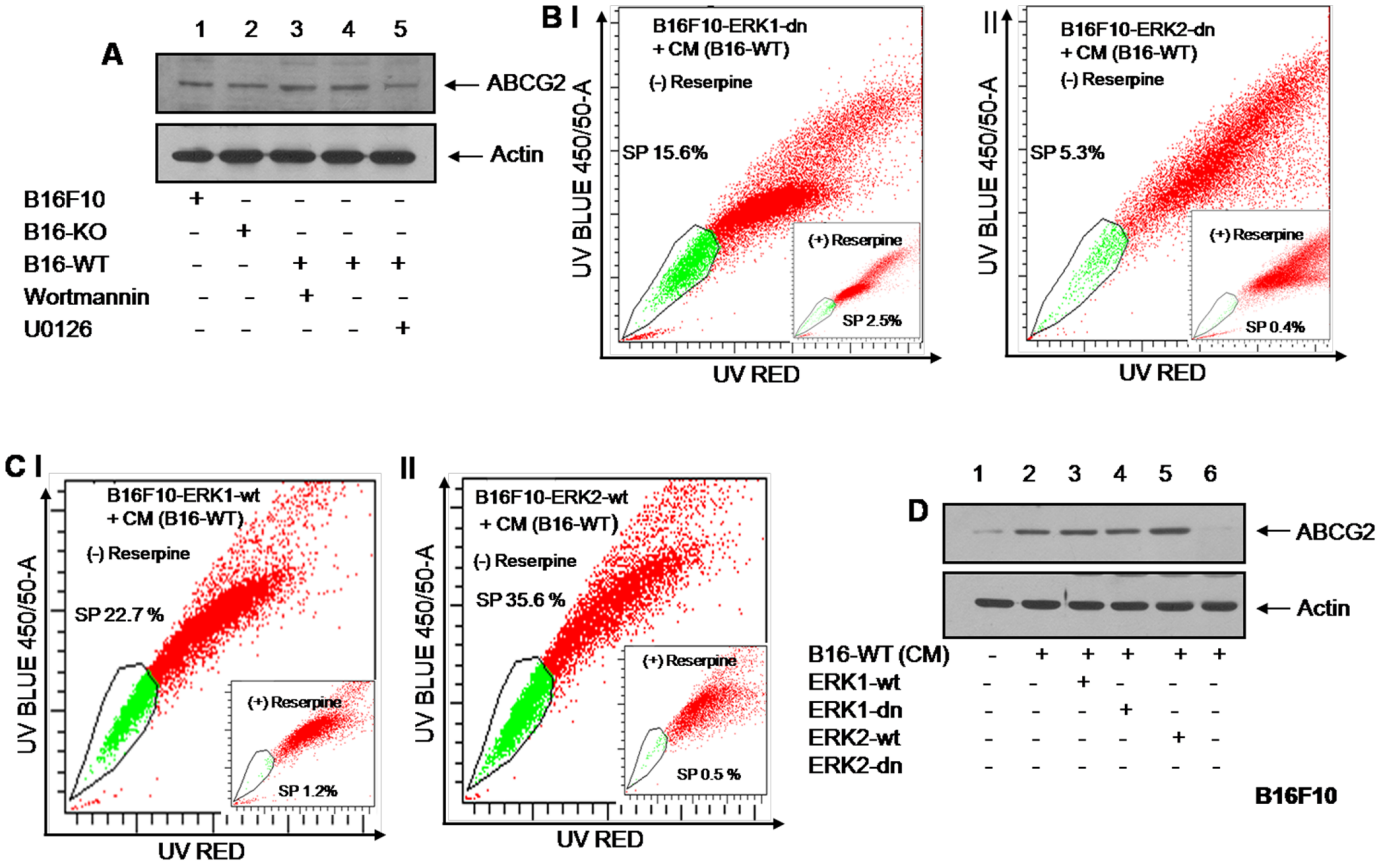
ERK2 but not ERK1 regulates SP phenotype in B16F10 cells in response to stromal OPN. (**A**) Western blot analysis of ABCG2 expression in the lysates of B16F10, B16-KO and B16-WT cells treated with either wortmannin or U0126. (**B**) *Panels I and II*, B16F10 cells, stably transfected with either ERK1-dn or ERK2-dn were treated with conditioned media of B16-WT cells, stained with Hoechst and SP phenotype was analyzed using flow cytometer. (**C**) *Panels I and II*, B16F10 cells stably transfected with either ERK1-wt or ERK2-wt were treated with CM collected from B16-WT cells, stained with Hoechst and SP phenotype was analyzed. *Inset:* control setup for SP analysis treated with reserpine for respective treatment group. (**D**) Analysis of ABCG2 expression from lysates of B16F10 cells stably transfected with ERK1-dn, ERK2-dn, ERK1-wt or ERK2-wt in presence of CM of B16-WT. Actin was used as loading control. All the figures are representative of three independent experiments exhibiting similar results.

B16-WT cells were treated with wortmannin or U0126 and ABCG2 expression was analyzed by western blot. The result suggested that inhibiting PI3-kinase signaling has no effect but blocking ERK signaling significantly reduced ABCG2 expression (Fig. 7A). This further confirms that stromal OPN enhanced ABCG2 expression through ERK signaling and regulate SP phenotype.

### ERK2 but not ERK1 Regulates SP Phenotype in Response to Stromal-OPN in B16F10 Cells

To further dissect ERK signaling in response to stromal-OPN in B16F10 cells, loss of function studies were performed. B16F10 cells were stably transfected with kinase negative ERK1 (ERK1-dn) and kinase negative ERK2 (ERK2-dn), and then treated with CM of B16-WT cells followed by staining with Hoechst dyes and analyzed for SP phenotype. Unexpectedly, drastic reduction in SP phenotype (5.3%) was observed in ERK2-dn transfected melanoma cells but not in ERK1-dn cells (15.6%) (Fig. 7B, panel I and II) as compared to B16F10 cells treated with CM of B16-WT (19.4%) (Fig. 6E) suggested that ERK2 plays an important role in regulation of SP phenomena in B16F10 cells.

To further confirm our observations, gain of function studies were performed with B16F10 cells stably transfected with ERK1-wt and ERK2-wt. These clones were treated with CM of B16-WT cells, stained with Hoechst dyes and analyzed for SP. Surprisingly, only 22.7% SP was observed in ERK1-wt transfected melanoma cell (Fig. 7C, panel I). In contrast, drastic increase in SP phenotype (35.6%) was observed in ERK2-wt transfected melanoma cells (Fig. 7C, panel II). We further sought to examine the expression of ABCG2 in these clones treated with conditioned media collected from B16-WT by western blot analysis. As expected, loss or gain of ERK1 function has no impact on ABCG2 expression. Gain of ERK2 function led to ABCG2 upregulation whereas loss of ERK2 function results in downregulation of ABCG2 expression (Fig. 7D). These results further suggested that stromal-OPN enriches SP phenotype through ERK2 dependent signaling.

## Discussion

In the current study, we have observed significantly reduced tumor growth in OPN^−/−^ mice as compared to OPN^+/+^ mice. Earlier Jessani *et al* have demonstrated that human breast cancer cells isolated from orthotopic xenograft tumor in SCID mice, exhibit profound differences in their enzyme activity profiles and showed enhanced tumor growth and metastasis [9]. Reduced tumor metastasis in bone and lung of OPN deficient mice has been shown earlier [47], [48]. Our *in vitro* data showed that B16-WT cells exhibit profound aggressive behavior compared to B16F10 or B16-KO cells. Consequently, we have proposed three hypotheses regarding such belligerent behavior of B16-WT cells: (i) stromal OPN might induce constitutive changes in melanoma cells resulting in enhanced activation of kinases and transcription factors and elevated expression of oncogenic molecules which in turn regulates the belligerent behavior of B16-WT cells; (ii) stromal-OPN might select the aggressive subpopulation from the heterogeneous parental B16F10 cells and produce a highly tumorigenic population of melanoma cells which exhibit enhanced belligerent behavior of tumor and (iii) stromal OPN might selectively promote the growth of cancer stem cell population of B16F10 cells and exhibit the aggressive behavior (Fig. S5).

Although researchers have earlier demonstrated that stromal OPN may regulate hematopoietic stem cells in mice model, its role on cancer stem cell has not been studied well [29]–[31]. We extended our study and checked the SP phenotype and found that B16F10 cells exhibit SP phenotype and stromal-OPN selectively enriches it in mice model. We further observed that B16-WT cells possess the capacity to regulate SP phenotype in B16F10 cells. Further we demonstrated that ERK2 but not ERK1 is capable of regulating the SP phenotype in B16F10 melanoma cells in response to stromal-OPN.

In conclusion, using *in vitro* and *in vivo* models, we have demonstrated, at least in part, the essential role of stroma derived OPN in regulation of melanoma growth, angiogenesis and metastasis. Moreover, we have delineated that stromal OPN regulates constitutive changes which resulted in acquisition of an aggressive or tumor initiating phenotype leading to rapid tumor growth, angiogenesis and metastasis. These results demonstrated that the expression profile of stromal-OPN may act as an early prognostic marker and suggested the intriguing possibility of stromal-OPN targeted therapy in cancer management.

## Supporting Information

Figure S1 (**A)** Isolated tumors from WT and KO mice were weighed, analyzed statistically and represented in the form of bar graph. Mean±SD; *, P<0.001. (**B)** Photographs of primary culture from the tumor tissue derived from subcutaneous injection of B16F10 cells into OPN^+/+^ and OPN^−/−^ mice. (**C)** Wound closure was quantified using Image Pro-plus software, analyzed statistically and represented graphically. Bars, mean±SEM; *, P<0.001 vs. control. (**D)** Melanoma cells (B16F10, B16-WT or B16-KO) were plated on upper chamber of transwell whereas lower chamber were filled with medium containing 2% FBS. Migrated cells in the opposite side of upper chamber were fixed, stained with Giemsa and photographed. (**E)** The tubes formed were counted using Image Pro-plus software, analyzed statistically and represented graphically. Bars, mean±SEM; *, P<0.002 vs. control.
(TIF)

Figure S2 (**A)** and (**B)** Melanoma cells were seeded in the lower chamber whereas HUVEC (1×10^5^) were plated in upper chamber of modified Boyden chamber or matrigel coated invasion chamber and incubated for 12 h. Migrated or invaded HUVEC on opposite side of upper chamber were stained with Giemsa and photographed. (**C)** Isolated tumors generated in KO mice derived from B16F10, B16-WT and B16-KO cells were weighed, analyzed statistically and represented in the form of bar graph. Bars, mean±SD; *, P<0.001 vs. control. (**D)** Sorted SP and non-SP cells were seeded on matrigel coated plate and incubated for 10 days. Colonies formed were imaged, counted, analyzed statistically and represented graphically. Bars, mean±SEM; *, P<0.012. (**E)** Sorted SP cells (1×10^3^) were injected into the OPN^+/+^ and OPN^−/−^ mice and kept for 5 weeks. Mice were sacrificed and tumors were collected and photographed.
(TIF)

Figure S3 **Analysis of SP phenotype from B16-KO-GFP cells.** (**A–F)** B16-KO-GFP cells were stained with Hoechst in absence or presence of reserpine and analyzed for SP phenotype.
(TIF)

Figure S4 **Analysis of SP phenotype from B16-WT-GFP cells.** (**A–F)** B16-WT-GFP cells were stained with Hoechst in absence or presence of reserpine and analyzed for SP phenotype.
(TIF)

Figure S5 Schematic diagram of mechanism involved in host/stromal OPN induced melanoma progression and angiogenesis.
(TIF)
