# Supplementary figures and images for: Functional Characterization of Stromal Osteopontin in Melanoma Progression and Metastasis
Source: PLoS One. 2013 Jul 23;8(7):e69116. doi: 10.1371/journal.pone.0069116 (PMC3720680; doi:10.1371/journal.pone.0069116)

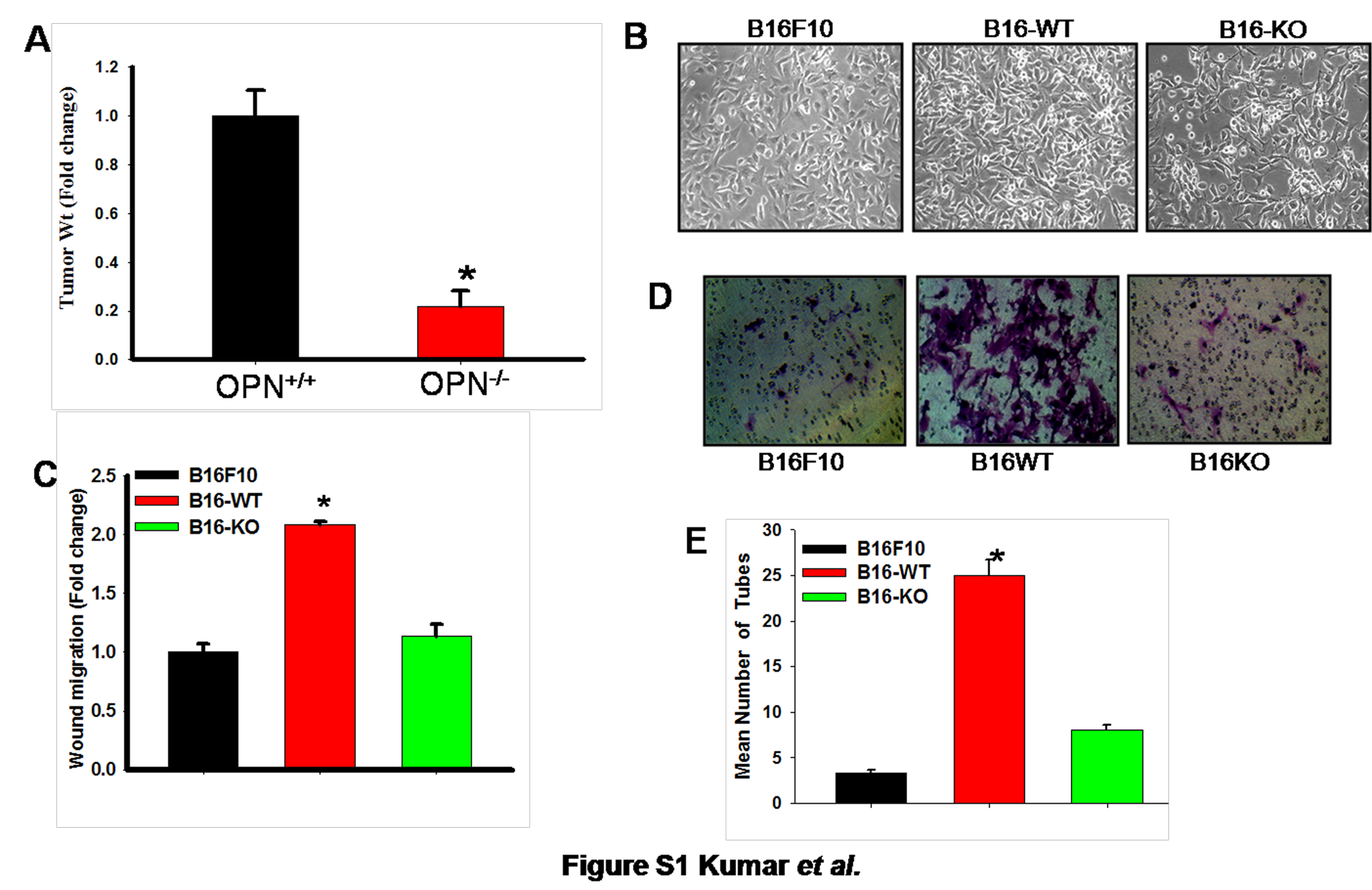

Supplement: Figure S1 — (A) Isolated tumors from WT and KO mice were weighed, analyzed statistically and represented in the form of bar graph. Mean±SD; *, P<0.001. (B) Photographs of primary culture from the tumor tissue derived from subcutaneous injection of B16F10 cells into OPN+/+ and OPN−/− mice. (C) Wound closure was quantified using Image Pro-plus software, analyzed statistically and represented graphically. Bars, mean±SEM; *, P<0.001 vs. control. (D) Melanoma cells (B16F10, B16-WT or B16-KO) were plated on upper chamber of transwell whereas lower chamber were filled with medium containing 2% FBS. Migrated cells in the opposite side of upper chamber were fixed, stained with Giemsa and photographed. (E) The tubes formed were counted using Image Pro-plus software, analyzed statistically and represented graphically. Bars, mean±SEM; *, P<0.002 vs. control. (TIF) [file pone.0069116.s001.tif]

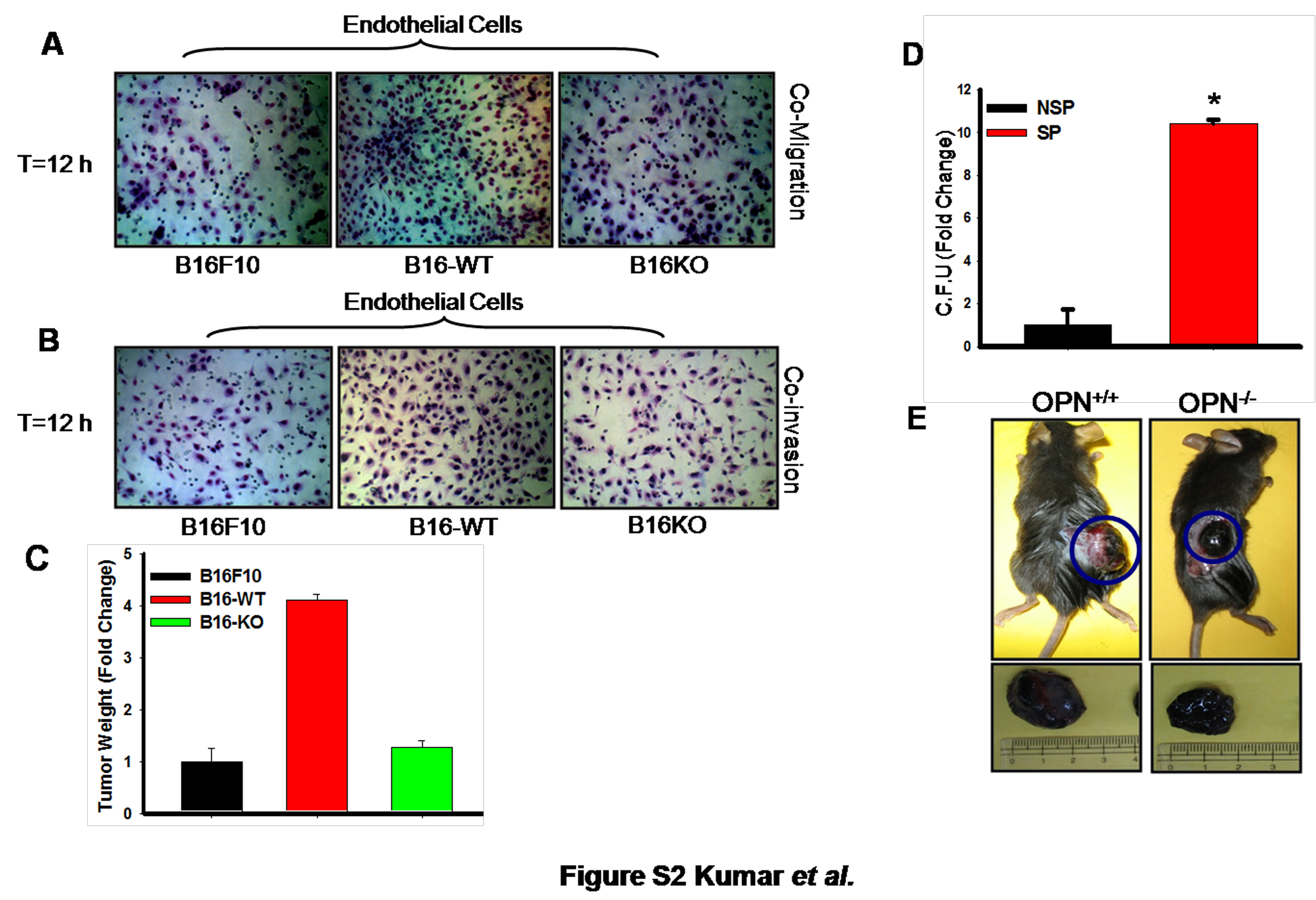

Supplement: Figure S2 — (A) and (B) Melanoma cells were seeded in the lower chamber whereas HUVEC (1×10^5^) were plated in upper chamber of modified Boyden chamber or matrigel coated invasion chamber and incubated for 12 h. Migrated or invaded HUVEC on opposite side of upper chamber were stained with Giemsa and photographed. (C) Isolated tumors generated in KO mice derived from B16F10, B16-WT and B16-KO cells were weighed, analyzed statistically and represented in the form of bar graph. Bars, mean±SD; *, P<0.001 vs. control. (D) Sorted SP and non-SP cells were seeded on matrigel coated plate and incubated for 10 days. Colonies formed were imaged, counted, analyzed statistically and represented graphically. Bars, mean±SEM; *, P<0.012. (E) Sorted SP cells (1×10^3^) were injected into the OPN+/+ and OPN−/− mice and kept for 5 weeks. Mice were sacrificed and tumors were collected and photographed. (TIF) [file pone.0069116.s002.tif]

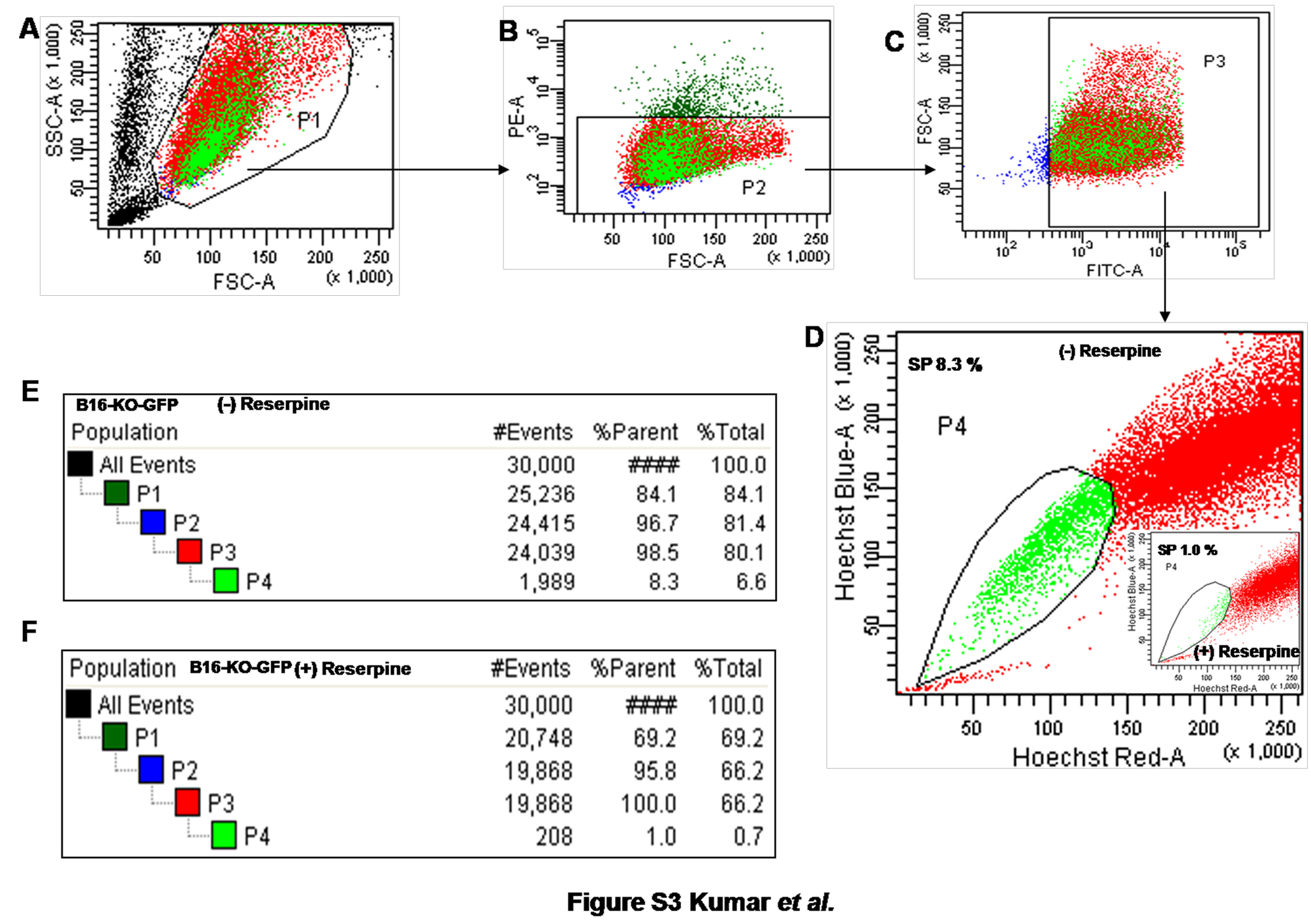

Supplement: Figure S3 — Analysis of SP phenotype from B16-KO-GFP cells. (A–F) B16-KO-GFP cells were stained with Hoechst in absence or presence of reserpine and analyzed for SP phenotype. (TIF) [file pone.0069116.s003.tif]

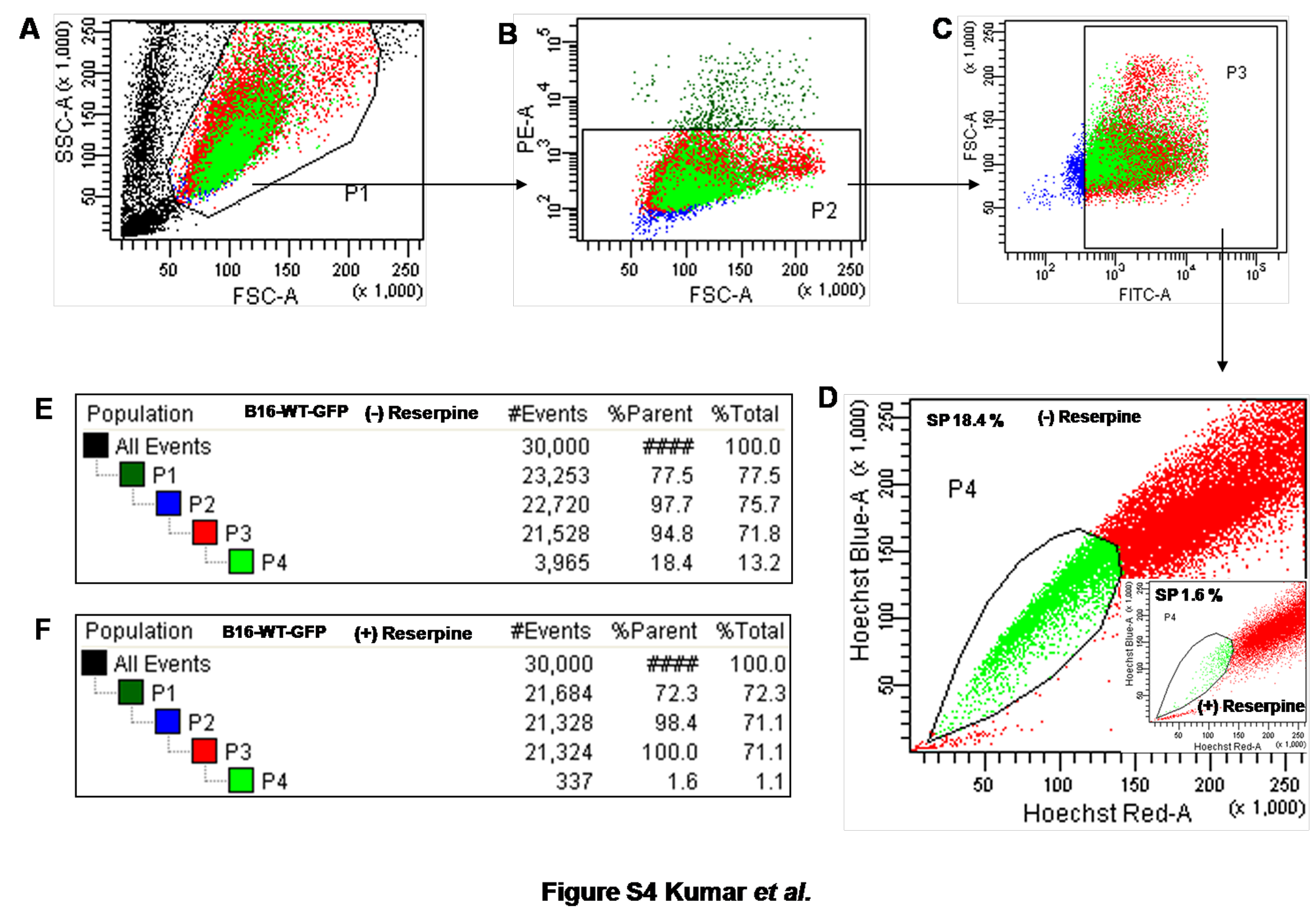

Supplement: Figure S4 — Analysis of SP phenotype from B16-WT-GFP cells. (A–F) B16-WT-GFP cells were stained with Hoechst in absence or presence of reserpine and analyzed for SP phenotype. (TIF) [file pone.0069116.s004.tif]

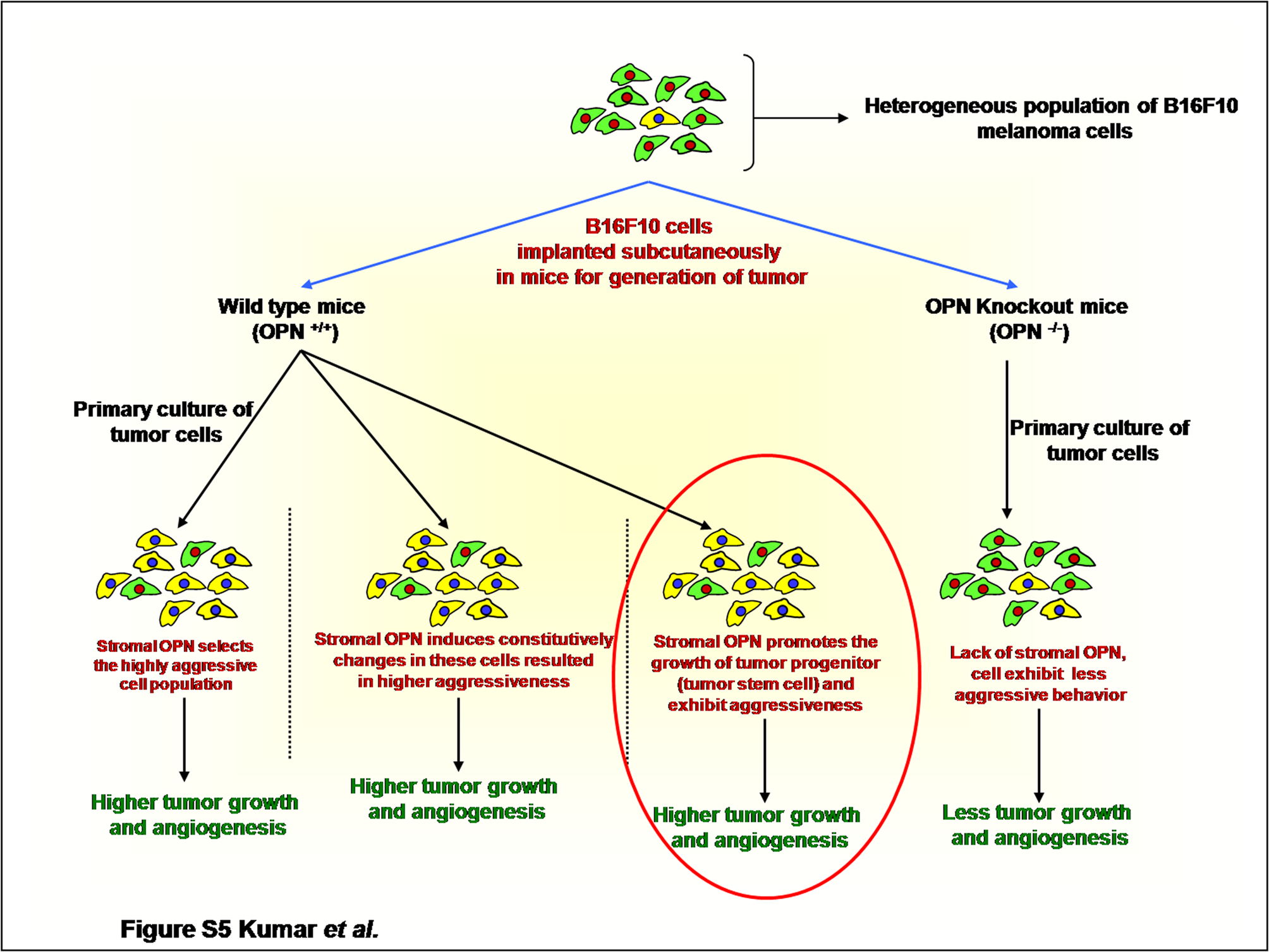

Supplement: Figure S5 — Schematic diagram of mechanism involved in host/stromal OPN induced melanoma progression and angiogenesis. (TIF) [file pone.0069116.s005.tif]
